# Di-μ_2_-chlorido-bis­{chlorido­[2,4,6-tris­(pyridin-2-yl)-1,3,5-triazine-κ^3^ *N* ^2^,*N* ^1^,*N* ^6^]nickel(II)}

**DOI:** 10.1107/S2414314621000936

**Published:** 2021-01-29

**Authors:** Kwang Ha

**Affiliations:** a Chonnam National University, School of Chemical Engineering, Research Institute of Catalysis, Gwangju, Republic of Korea; University Koblenz-Landau, Germany

**Keywords:** crystal structure, nickel(II) complex, 2,4,6-tri-2-pyridyl-1,3,5-triazine, dinuclear complex

## Abstract

In the title compound, [Ni_2_Cl_4_(C_18_H_12_N_6_)_2_], the Ni^II^ ions are hexa-coordinated in a distorted octa­hedral coordination environment defined by three N atoms of the tridentate 2,4,6-tri-2-pyridyl-1,3,5-triazine ligand and three Cl^−^ anions in a meridional geometry. The two Ni^II^ ions are bridged by two Cl anionic ligands, thereby forming a dinuclear complex. A crystallographic centre of inversion is located at the centroid of the Ni_2_Cl_2_ ring.

## Abstract 2

The Ni^II^ ions both show a distorted N_3_Cl_3_ octa­hedral coordination environment defined by three N atoms of the tridentate 2,4,6-tri-2-pyridyl-1,3,5-triazine ligand, two bridging Cl^−^ ligands and a Cl^−^ anion.

## Graphical abstract

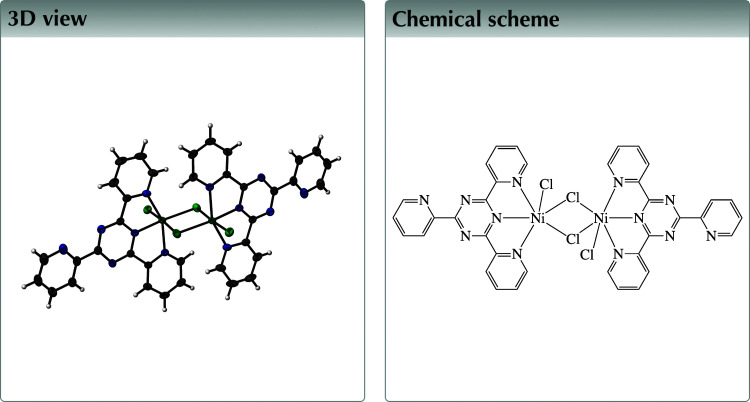

## Structure description

With reference to the title compound, [Ni_2_Cl_4_(tptz)_2_] (tptz = 2,4,6-tri-2-pyridyl-1,3,5-triazine), the crystal structures of related chlorido Ni^II^ complexes [NiCl_2_(tptz)(CH_3_OH)] (Hadadzadeh *et al.*, 2012 ▸), [NiCl(H-tptz)(H_2_O)_2_]Cl_2_·2H_2_O (Zibaseresht & Hartshorn, 2005 ▸) and [NiCl_2_(py)(tptz)] (py = pyridine) (Ha, 2019 ▸) have been determined previously.

**Figure 1:**
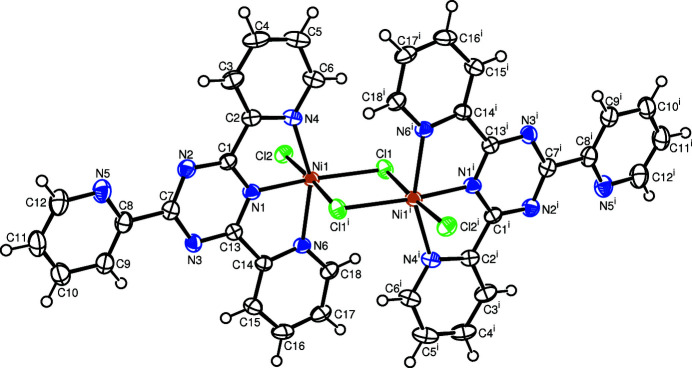
Mol­ecular structure of the title compound showing the atom labelling and displacement ellipsoids drawn at the 50% probability level for non-H atoms. Symmetry code: (i) −*x*, −*y* + 1, −*z*.

In the complex, the two Ni^II^ cations are bridged by two chlorido ligands to form a dinuclear complex. A crystallographic centre of inversion is located at the centroid of the Ni_2_Cl_2_ ring. The asymmetric unit therefore contains one half of the complex (Fig. 1 ▸). Each Ni^II^ atom is hexa-coordinated in a considerably distorted octa­hedral coordination environment defined by three N atoms of the tridentate tptz ligand, two bridging Cl^−^ ligands and one terminal Cl^−^ anion. The main contributions to the distortion are the tight N—Ni—N chelating angles [N1—Ni1—N4 = 76.96 (6)° and N1—Ni1—N6 = 77.52 (6)°] and the chlorido bridges, which result in a non-linear *trans* arrangement of the N4—Ni1—N6 and N1—Ni1—Cl axes [N4—Ni1—N6 = 154.46 (6)° and N1—Ni1—Cl1 = 169.87 (5)°]. On the other hand the Cl2—Ni1—Cl1^i^ axis (symmetry code: (i) −*x*, −*y* + 1, −*z*) is almost linear [Cl2—Ni1—Cl1^i^ = 176.25 (2)°]. The Ni—N(pyrid­yl) bonds [Ni1—N4/N6 = 2.130 (2) and 2.129 (2) Å] are slightly longer than the Ni—N(triazine) bond [Ni1—N1 = 1.970 (2) Å]. The three Ni—Cl bond lengths are somewhat different [Ni1—Cl1^i^ = 2.5812 (5), Ni1—Cl1 = 2.3326 (5) and Ni1—Cl2 = 2.3538 (5) Å]. The two pyridyl rings that coordinat to the Ni^II^ atom are located approximately parallel to the respective triazine ring, making dihedral angles of 4.51 (6) and 4.95 (6)°, respectively. The dihedral angle between the non-coordinating pyridyl substituent and the triazine ring is 7.56 (6)°.

**Figure 2:**
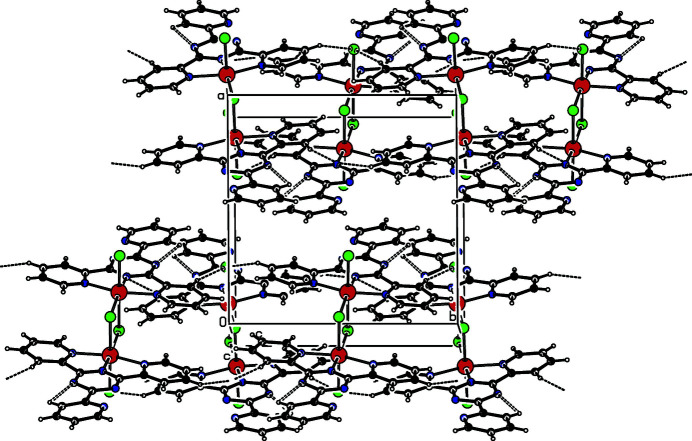
Crystal structure of the title compound showing π–π inter­actions as well as weak C–H⋯Cl hydrogen bonds.

The complex displays numerous inter­molecular π–π inter­actions between adjacent six-membered rings. For *Cg*1 (the centroid of ring N5/C8–C12) and *Cg*2^ii^ [the centroid of ring N6/C14–C18; symmetry code: (ii) *x*, −*y* + 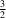 , *z* − 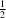 ], the centroid–centroid distance is 4.138 (1) Å and the dihedral angle between the ring planes is 5.44 (10)°. In addition, the complex reveals inter­molecular C—H⋯Cl hydrogen bonds with distances of 2.773 (3)–3.605 (2) Å between the donor and acceptor atoms, to stabilize the crystal structure (Table 1 ▸, Fig. 2 ▸).

## Synthesis and crystallization

To a solution of NiCl_2_·6 H_2_O (0.2670 g, 1.123 mmol) in ethanol (30 ml) was added 2,4,6-tri-2-pyridyl-1,3,5-triazine (0.2814 g, 0.901 mmol). The solution was stirred for 12 h at room temperature. The formed precipitate was separated by filtration, washed with ethanol and acetone, and dried at 323 K, to give a pale-green powder (0.3363 g, 84%). Brown crystals suitable for X-ray analysis were obtained by slow evaporation from a dimethyl sulfoxide (DMSO) solution at 363 K.

## Refinement

Crystal data, data collection and structure refinement details are summarized in Table 2 ▸. The remaining maximum (0.33 e Å^−3^) and minimum (−0.20 e Å^−3^) electron density in the difference Fourier map are located 0.73 and 1.28 Å, respectively, from atoms C2 and C9.

## Supplementary Material

Crystal structure: contains datablock(s) I. DOI: 10.1107/S2414314621000936/im4011sup1.cif

Structure factors: contains datablock(s) I. DOI: 10.1107/S2414314621000936/im4011Isup2.hkl

CCDC reference: 2058987

Additional supporting information:  crystallographic information; 3D view; checkCIF report

## Figures and Tables

**Table 1:** Hydrogen-bond geometry (Å, °)

*D*—H⋯*A*	*D*—H	H⋯*A*	*D*⋯*A*	*D*—H⋯*A*
C4—H4⋯Cl2^i^	0.94	2.76	3.605 (2)	150
C15—H15⋯Cl2^ii^	0.94	2.57	3.471 (2)	162

Symmetry codes: (i) 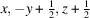 ; (ii) 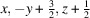 .

**Table 2:** Experimental details

Crystal data	
Chemical formula	[Ni_2_Cl_4_(C_18_H_12_N_6_)_2_]
*M*_r_	883.89
Crystal system, space group	Monoclinic, *P*2_1_/*c*
Temperature (K)	223
*a*, *b*, *c* (Å)	13.0130 (4), 12.8275 (4), 11.0153 (3)
β (°)	106.5083 (11)
*V* (Å^3^)	1762.93 (9)
*Z*	2
Radiation type	Mo *K*α
μ (mm^−1^)	1.42
Crystal size (mm)	0.25 × 0.15 × 0.13

Data collection	
Diffractometer	PHOTON 100 CMOS detector
Absorption correction	Multi-scan (*SADABS*; Bruker, 2016 ▸)
*T*_min_, *T*_max_	0.660, 0.745
No. of measured, independent and observed [*I* > 2σ(*I*)] reflections	48040, 3487, 2828
*R*_int_	0.059
(sin θ/λ)_max_ (Å^−1^)	0.618

Refinement	
*R*[*F* ^2^ > 2σ(*F* ^2^)], *wR*(*F* ^2^), *S*	0.028, 0.067, 1.06
No. of reflections	3487
No. of parameters	244
H-atom treatment	H-atom parameters constrained
Δρ_max_, Δρ_min_ (e Å^−3^)	0.33, −0.20

Computer programs: *APEX2* and *SAINT* (Bruker, 2016 ▸), *SHELXT2014/7* (Sheldrick, 2015*a* ▸), *SHELXL2014/7* (Sheldrick, 2015*b* ▸), *ORTEP-3 for Windows* (Farrugia, 2012 ▸) and *PLATON* (Spek, 2020 ▸).

## full crystallographic data

### Crystal data

**Table:**

[Ni_2_Cl_4_(C_18_H_12_N_6_)_2_]	*F*(000) = 896
*M**_r_* = 883.89	*D*_x_ = 1.665 Mg m^−^^3^
Monoclinic, *P*2_1_/*c*	Mo *K*α radiation, λ = 0.71073 Å
*a* = 13.0130 (4) Å	Cell parameters from 9969 reflections
*b* = 12.8275 (4) Å	θ = 2.3–28.2°
*c* = 11.0153 (3) Å	µ = 1.42 mm^−^^1^
β = 106.5083 (11)°	*T* = 223 K
*V* = 1762.93 (9) Å^3^	Block, brown
*Z* = 2	0.25 × 0.15 × 0.13 mm

### Data collection

**Table:**

PHOTON 100 CMOS detector diffractometer	2828 reflections with *I* > 2σ(*I*)
Radiation source: sealed tube	*R*_int_ = 0.059
φ and ω scans	θ_max_ = 26.1°, θ_min_ = 2.3°
Absorption correction: multi-scan (SADABS; Bruker, 2016)	*h* = −16→16
*T*_min_ = 0.660, *T*_max_ = 0.745	*k* = −15→15
48040 measured reflections	*l* = −13→13
3487 independent reflections	

### Refinement

**Table:**

Refinement on *F*^2^	Primary atom site location: structure-invariant direct methods
Least-squares matrix: full	Secondary atom site location: difference Fourier map
*R*[*F*^2^ > 2σ(*F*^2^)] = 0.028	Hydrogen site location: inferred from neighbouring sites
*wR*(*F*^2^) = 0.067	H-atom parameters constrained
*S* = 1.06	*w* = 1/[σ^2^(*F*_o_^2^) + (0.0314*P*)^2^ + 0.698*P*] where *P* = (*F*_o_^2^ + 2*F*_c_^2^)/3
3487 reflections	(Δ/σ)_max_ = 0.001
244 parameters	Δρ_max_ = 0.33 e Å^−^^3^
0 restraints	Δρ_min_ = −0.20 e Å^−^^3^

### Special details

**Table:**

Geometry. All esds (except the esd in the dihedral angle between two l.s. planes) are estimated using the full covariance matrix. The cell esds are taken into account individually in the estimation of esds in distances, angles and torsion angles; correlations between esds in cell parameters are only used when they are defined by crystal symmetry. An approximate (isotropic) treatment of cell esds is used for estimating esds involving l.s. planes.
Refinement. Hydrogen atoms on C atoms were positioned geometrically and allowed to ride on their respective parent atoms: C—H = 0.94 Å and *U*_iso_(H) = 1.2 *U*_eq_(C).

### Fractional atomic coordinates and isotropic or equivalent isotropic displacement parameters (Å^2^)

**Table:**

	*x*	*y*	*z*	*U*_iso_*/*U*_eq_	
Ni1	0.14246 (2)	0.51866 (2)	0.04761 (2)	0.02427 (9)	
Cl1	0.01562 (4)	0.48538 (4)	−0.14563 (4)	0.02986 (13)	
Cl2	0.29409 (4)	0.52462 (4)	−0.02845 (5)	0.03079 (13)	
N1	0.22767 (13)	0.54763 (12)	0.22294 (15)	0.0248 (4)	
N2	0.32398 (14)	0.48592 (13)	0.42305 (15)	0.0287 (4)	
N3	0.29552 (13)	0.66875 (13)	0.38378 (15)	0.0276 (4)	
N4	0.18958 (13)	0.36721 (12)	0.12204 (15)	0.0267 (4)	
N5	0.42794 (15)	0.52970 (14)	0.66906 (16)	0.0374 (4)	
N6	0.13351 (12)	0.68398 (12)	0.05789 (14)	0.0247 (4)	
C1	0.26891 (15)	0.47112 (15)	0.30292 (18)	0.0246 (4)	
C2	0.24779 (16)	0.36589 (15)	0.24522 (18)	0.0256 (4)	
C3	0.28603 (17)	0.27542 (16)	0.3092 (2)	0.0339 (5)	
H3	0.3234	0.2769	0.3958	0.041*	
C4	0.26843 (19)	0.18244 (17)	0.2435 (2)	0.0409 (6)	
H4	0.2930	0.1192	0.2848	0.049*	
C5	0.21447 (19)	0.18388 (17)	0.1169 (2)	0.0413 (6)	
H5	0.2045	0.1219	0.0696	0.050*	
C6	0.17494 (17)	0.27710 (15)	0.0595 (2)	0.0324 (5)	
H6	0.1364	0.2769	−0.0268	0.039*	
C7	0.33706 (15)	0.58634 (16)	0.45819 (18)	0.0278 (4)	
C8	0.39884 (16)	0.61154 (17)	0.59008 (19)	0.0302 (5)	
C9	0.42105 (16)	0.71422 (18)	0.6268 (2)	0.0338 (5)	
H9	0.3990	0.7689	0.5684	0.041*	
C10	0.47666 (17)	0.73425 (19)	0.7519 (2)	0.0394 (6)	
H10	0.4937	0.8030	0.7802	0.047*	
C11	0.50621 (18)	0.6520 (2)	0.8335 (2)	0.0426 (6)	
H11	0.5435	0.6635	0.9190	0.051*	
C12	0.48059 (18)	0.5520 (2)	0.7888 (2)	0.0438 (6)	
H12	0.5015	0.4964	0.8462	0.053*	
C13	0.23995 (15)	0.64467 (15)	0.26684 (18)	0.0237 (4)	
C14	0.18376 (15)	0.72372 (15)	0.17355 (18)	0.0238 (4)	
C15	0.18125 (16)	0.82788 (15)	0.2024 (2)	0.0301 (5)	
H15	0.2164	0.8528	0.2839	0.036*	
C16	0.12571 (17)	0.89494 (16)	0.1083 (2)	0.0350 (5)	
H16	0.1212	0.9663	0.1255	0.042*	
C17	0.07695 (17)	0.85609 (16)	−0.0108 (2)	0.0336 (5)	
H17	0.0406	0.9009	−0.0766	0.040*	
C18	0.08231 (15)	0.75012 (16)	−0.03215 (19)	0.0287 (5)	
H18	0.0485	0.7239	−0.1134	0.034*	

### Atomic displacement parameters (Å^2^)

**Table:**

	*U*^11^	*U*^22^	*U*^33^	*U*^12^	*U*^13^	*U*^23^
Ni1	0.03109 (15)	0.01883 (14)	0.02120 (14)	0.00130 (10)	0.00470 (11)	0.00041 (10)
Cl1	0.0330 (3)	0.0344 (3)	0.0216 (2)	−0.0031 (2)	0.0068 (2)	−0.0029 (2)
Cl2	0.0314 (3)	0.0313 (3)	0.0298 (3)	0.0037 (2)	0.0088 (2)	0.0046 (2)
N1	0.0300 (9)	0.0208 (8)	0.0235 (8)	0.0015 (7)	0.0073 (7)	0.0012 (7)
N2	0.0311 (9)	0.0288 (9)	0.0254 (9)	0.0020 (7)	0.0066 (7)	0.0031 (7)
N3	0.0288 (9)	0.0281 (9)	0.0239 (9)	−0.0008 (7)	0.0044 (7)	−0.0001 (7)
N4	0.0331 (9)	0.0217 (9)	0.0280 (9)	−0.0003 (7)	0.0130 (7)	−0.0009 (7)
N5	0.0392 (11)	0.0432 (11)	0.0257 (9)	−0.0010 (9)	0.0028 (8)	0.0043 (8)
N6	0.0283 (9)	0.0215 (9)	0.0246 (9)	0.0013 (7)	0.0079 (7)	0.0015 (7)
C1	0.0256 (10)	0.0243 (10)	0.0249 (10)	0.0028 (8)	0.0088 (8)	0.0034 (8)
C2	0.0301 (11)	0.0237 (10)	0.0260 (10)	0.0024 (8)	0.0129 (8)	0.0027 (8)
C3	0.0400 (12)	0.0304 (12)	0.0350 (12)	0.0078 (9)	0.0165 (10)	0.0107 (10)
C4	0.0541 (15)	0.0222 (11)	0.0527 (15)	0.0098 (10)	0.0253 (12)	0.0115 (10)
C5	0.0569 (15)	0.0214 (11)	0.0533 (15)	−0.0005 (10)	0.0280 (12)	−0.0030 (10)
C6	0.0440 (13)	0.0241 (11)	0.0332 (12)	−0.0018 (9)	0.0177 (10)	−0.0026 (9)
C7	0.0258 (11)	0.0324 (11)	0.0257 (10)	0.0007 (9)	0.0081 (8)	0.0014 (9)
C8	0.0259 (11)	0.0396 (13)	0.0252 (10)	0.0003 (9)	0.0073 (9)	0.0018 (9)
C9	0.0294 (11)	0.0392 (13)	0.0307 (11)	0.0010 (9)	0.0051 (9)	0.0000 (10)
C10	0.0309 (12)	0.0491 (15)	0.0368 (13)	−0.0024 (11)	0.0075 (10)	−0.0112 (11)
C11	0.0362 (13)	0.0603 (16)	0.0269 (12)	−0.0022 (12)	0.0019 (10)	−0.0053 (11)
C12	0.0429 (14)	0.0539 (15)	0.0289 (12)	−0.0010 (12)	0.0012 (10)	0.0068 (11)
C13	0.0239 (10)	0.0241 (10)	0.0242 (10)	0.0001 (8)	0.0083 (8)	0.0012 (8)
C14	0.0252 (10)	0.0213 (10)	0.0262 (10)	−0.0006 (8)	0.0092 (8)	−0.0001 (8)
C15	0.0347 (12)	0.0236 (11)	0.0322 (11)	−0.0021 (9)	0.0095 (9)	−0.0030 (9)
C16	0.0401 (13)	0.0187 (11)	0.0492 (14)	0.0022 (9)	0.0174 (11)	0.0017 (10)
C17	0.0349 (12)	0.0252 (11)	0.0414 (13)	0.0069 (9)	0.0119 (10)	0.0106 (10)
C18	0.0298 (11)	0.0291 (11)	0.0277 (11)	0.0039 (9)	0.0090 (9)	0.0058 (8)

### Geometric parameters (Å, º)

**Table:**

Ni1—N1	1.9700 (16)	C4—C5	1.372 (3)
Ni1—N6	2.1286 (16)	C4—H4	0.9400
Ni1—N4	2.1300 (16)	C5—C6	1.382 (3)
Ni1—Cl1	2.3326 (5)	C5—H5	0.9400
Ni1—Cl2	2.3538 (5)	C6—H6	0.9400
Ni1—Cl1^i^	2.5812 (5)	C7—C8	1.482 (3)
Cl1—Ni1^i^	2.5811 (5)	C8—C9	1.384 (3)
N1—C1	1.326 (2)	C9—C10	1.387 (3)
N1—C13	1.329 (2)	C9—H9	0.9400
N2—C1	1.327 (3)	C10—C11	1.368 (3)
N2—C7	1.342 (3)	C10—H10	0.9400
N3—C13	1.322 (2)	C11—C12	1.381 (3)
N3—C7	1.353 (3)	C11—H11	0.9400
N4—C6	1.331 (3)	C12—H12	0.9400
N4—C2	1.353 (2)	C13—C14	1.481 (3)
N5—C12	1.333 (3)	C14—C15	1.376 (3)
N5—C8	1.347 (3)	C15—C16	1.382 (3)
N6—C18	1.331 (2)	C15—H15	0.9400
N6—C14	1.355 (2)	C16—C17	1.379 (3)
C1—C2	1.484 (3)	C16—H16	0.9400
C2—C3	1.375 (3)	C17—C18	1.385 (3)
C3—C4	1.380 (3)	C17—H17	0.9400
C3—H3	0.9400	C18—H18	0.9400
			
N1—Ni1—N6	77.52 (6)	C4—C5—H5	120.2
N1—Ni1—N4	76.96 (6)	C6—C5—H5	120.2
N6—Ni1—N4	154.46 (6)	N4—C6—C5	122.3 (2)
N1—Ni1—Cl1	169.87 (5)	N4—C6—H6	118.8
N6—Ni1—Cl1	101.28 (4)	C5—C6—H6	118.8
N4—Ni1—Cl1	103.65 (5)	N2—C7—N3	125.40 (18)
N1—Ni1—Cl2	92.73 (5)	N2—C7—C8	118.76 (18)
N6—Ni1—Cl2	92.90 (4)	N3—C7—C8	115.81 (18)
N4—Ni1—Cl2	89.31 (5)	N5—C8—C9	123.76 (19)
Cl1—Ni1—Cl2	97.384 (19)	N5—C8—C7	115.95 (18)
N1—Ni1—Cl1^i^	83.52 (5)	C9—C8—C7	120.27 (19)
N6—Ni1—Cl1^i^	86.30 (4)	C8—C9—C10	118.3 (2)
N4—Ni1—Cl1^i^	89.84 (5)	C8—C9—H9	120.9
Cl1—Ni1—Cl1^i^	86.370 (18)	C10—C9—H9	120.9
Cl2—Ni1—Cl1^i^	176.246 (19)	C11—C10—C9	118.7 (2)
Ni1—Cl1—Ni1^i^	93.631 (18)	C11—C10—H10	120.7
C1—N1—C13	117.87 (17)	C9—C10—H10	120.7
C1—N1—Ni1	121.36 (13)	C10—C11—C12	119.2 (2)
C13—N1—Ni1	120.64 (13)	C10—C11—H11	120.4
C1—N2—C7	114.41 (17)	C12—C11—H11	120.4
C13—N3—C7	114.93 (17)	N5—C12—C11	123.8 (2)
C6—N4—C2	117.72 (17)	N5—C12—H12	118.1
C6—N4—Ni1	127.55 (14)	C11—C12—H12	118.1
C2—N4—Ni1	114.53 (12)	N3—C13—N1	123.33 (18)
C12—N5—C8	116.3 (2)	N3—C13—C14	122.80 (17)
C18—N6—C14	117.79 (17)	N1—C13—C14	113.86 (16)
C18—N6—Ni1	128.22 (13)	N6—C14—C15	123.07 (18)
C14—N6—Ni1	113.93 (12)	N6—C14—C13	113.96 (16)
N1—C1—N2	123.94 (18)	C15—C14—C13	122.96 (18)
N1—C1—C2	113.45 (17)	C14—C15—C16	118.22 (19)
N2—C1—C2	122.61 (17)	C14—C15—H15	120.9
N4—C2—C3	122.83 (19)	C16—C15—H15	120.9
N4—C2—C1	113.64 (16)	C17—C16—C15	119.33 (19)
C3—C2—C1	123.51 (18)	C17—C16—H16	120.3
C2—C3—C4	118.6 (2)	C15—C16—H16	120.3
C2—C3—H3	120.7	C16—C17—C18	119.0 (2)
C4—C3—H3	120.7	C16—C17—H17	120.5
C5—C4—C3	118.8 (2)	C18—C17—H17	120.5
C5—C4—H4	120.6	N6—C18—C17	122.54 (19)
C3—C4—H4	120.6	N6—C18—H18	118.7
C4—C5—C6	119.6 (2)	C17—C18—H18	118.7
			
C13—N1—C1—N2	−1.8 (3)	N2—C7—C8—C9	174.92 (19)
Ni1—N1—C1—N2	−177.77 (14)	N3—C7—C8—C9	−6.9 (3)
C13—N1—C1—C2	178.24 (16)	N5—C8—C9—C10	0.2 (3)
Ni1—N1—C1—C2	2.3 (2)	C7—C8—C9—C10	178.34 (18)
C7—N2—C1—N1	−1.2 (3)	C8—C9—C10—C11	−0.6 (3)
C7—N2—C1—C2	178.72 (17)	C9—C10—C11—C12	0.5 (3)
C6—N4—C2—C3	−4.1 (3)	C8—N5—C12—C11	−0.4 (3)
Ni1—N4—C2—C3	−179.45 (15)	C10—C11—C12—N5	0.0 (4)
C6—N4—C2—C1	174.13 (17)	C7—N3—C13—N1	−1.9 (3)
Ni1—N4—C2—C1	−1.2 (2)	C7—N3—C13—C14	176.77 (17)
N1—C1—C2—N4	−0.6 (2)	C1—N1—C13—N3	3.5 (3)
N2—C1—C2—N4	179.51 (17)	Ni1—N1—C13—N3	179.50 (14)
N1—C1—C2—C3	177.70 (18)	C1—N1—C13—C14	−175.29 (16)
N2—C1—C2—C3	−2.2 (3)	Ni1—N1—C13—C14	0.7 (2)
N4—C2—C3—C4	3.0 (3)	C18—N6—C14—C15	2.1 (3)
C1—C2—C3—C4	−175.10 (19)	Ni1—N6—C14—C15	−175.50 (15)
C2—C3—C4—C5	0.6 (3)	C18—N6—C14—C13	−179.03 (16)
C3—C4—C5—C6	−2.9 (3)	Ni1—N6—C14—C13	3.4 (2)
C2—N4—C6—C5	1.7 (3)	N3—C13—C14—N6	178.41 (17)
Ni1—N4—C6—C5	176.33 (16)	N1—C13—C14—N6	−2.8 (2)
C4—C5—C6—N4	1.8 (3)	N3—C13—C14—C15	−2.7 (3)
C1—N2—C7—N3	3.0 (3)	N1—C13—C14—C15	176.14 (18)
C1—N2—C7—C8	−179.11 (17)	N6—C14—C15—C16	−0.7 (3)
C13—N3—C7—N2	−1.5 (3)	C13—C14—C15—C16	−179.52 (18)
C13—N3—C7—C8	−179.46 (17)	C14—C15—C16—C17	−1.3 (3)
C12—N5—C8—C9	0.3 (3)	C15—C16—C17—C18	1.8 (3)
C12—N5—C8—C7	−177.95 (19)	C14—N6—C18—C17	−1.4 (3)
N2—C7—C8—N5	−6.8 (3)	Ni1—N6—C18—C17	175.71 (15)
N3—C7—C8—N5	171.38 (17)	C16—C17—C18—N6	−0.5 (3)

Symmetry code: (i) −*x*, −*y*+1, −*z*.

### Hydrogen-bond geometry (Å, º)

**Table:**

*D*—H···*A*	*D*—H	H···*A*	*D*···*A*	*D*—H···*A*
C4—H4···Cl2^ii^	0.94	2.76	3.605 (2)	150
C15—H15···Cl2^iii^	0.94	2.57	3.471 (2)	162

Symmetry codes: (ii) *x*, −*y*+1/2, *z*+1/2; (iii) *x*, −*y*+3/2, *z*+1/2.
